# Scavenging for Hydroxybenzoic Acids in *Cupriavidus necator*: Studying Ligand Sensitivity Using a Biosensor-Based Approach

**DOI:** 10.3390/biom16010157

**Published:** 2026-01-15

**Authors:** Ingrida Sabaliauske, Ernesta Augustiniene, Rizkallah Al Akiki Dit Al Mazraani, Monika Tamasauskaite, Naglis Malys

**Affiliations:** 1 Bioprocess Research Centre, Faculty of Chemical Technology, Kaunas University of Technology, Radvilėnų Street 19, LT-50254 Kaunas, Lithuania; 2 Department of Organic Chemistry, Faculty of Chemical Technology, Kaunas University of Technology, Radvilėnų Street 19, LT-50254 Kaunas, Lithuania

**Keywords:** transcription factor-based biosensor, hydroxybenzoic acid, 2-hydroxybenzoate, 4-hydroxybenzoate, *Cupriavidus necator*

## Abstract

The increasing demand for rapid identification of bacteria capable of degrading environmentally relevant organic compounds highlights the need for scalable and selective analytical tools. *Cupriavidus necator* catabolizes several hydroxybenzoic acids, including 2-hydroxybenzoate (salicylate, 2-HBA), 4-hydroxybenzoate (4-HBA), and 3-hydroxybenzoate (3-HBA), funneling them into central aromatic catabolism via monooxygenation to 2,5-dihydroxybenzoate (gentisate, 2,5-dHBA) and 3,4-dihydroxybenzoate (protocatechuate, 3,4-dHBA) followed by the oxidative cleavage reaction, enabling complete conversion to tricarboxylic acid (TCA) cycle intermediates. To quantify how readily *C. necator* is able to activate catabolic genes in response to hydroxybenzoic acid, an extracellular ligand, we applied an approach centered on a transcription-factor (TF)-based biosensor that combines ligand-bound regulator activity with a fluorescent reporter. This approach allowed to evaluate the ligand sensitivity by determining gene activation threshold AC_min_ and half-maximal effective concentration EC_50_. Amongst studied hydroxybenzoic acids, 2-HBA and 4-HBA sensors from *C. necator* showed very low thresholds 4.8 and 2.4 μM and EC_50_ values of 19.91 and 13.06 μM, indicating high sensitivity to these compounds and implicating a scavenging characteristic of associated catabolism. This study shows that the TF-based-biosensor approach applied for mapping functional sensing ranges of hydroxybenzoates combined with the research and informatics of catabolism can advance our understanding of how gene expression regulation systems have evolved to respond differentially to the availability and concentration of carbon sources. Furthermore, it can inform metabolic engineering strategies in the prevention of premature pathway activation or in predicting competitive substrate hierarchies in complex mixed environments.

## 1. Introduction

Hydroxybenzoic acids (HBAs) are important aromatic compounds with broad industrial and environmental relevance, functioning as precursors in cosmetics, pharmaceuticals, food, and agricultural industries, as well as intermediates in the synthesis of various fine chemicals [1,2,3,4]. HBAs form a multi-million-dollar sector that is experiencing sustained growth [1]. For example, the global 4-HBA market was valued at USD 213 million in 2025 and is projected to reach USD 399.8 million by 2035 with an annual growth of 6.5% from 2025 to 2035 [5].

HBAs are important metabolic intermediates and substrates in the bacterial degradation of aromatic compounds and they have attracted significant attention in both the biotechnological production and bioremediation of environmental pollutants [1,2]. These applications underscore the biological and biotechnological relevance of HBAs and highlight the need for sensitive, well-characterized tools capable of monitoring their intracellular levels for rapid strain development and efficient biosynthesis, as well as for detection in complex environments.

Bacteria have evolved regulatory systems dedicated to detecting aromatic compounds and coordinating metabolic pathways for their assimilation [6,7]. Among these organisms, *Cupriavidus necator* H16 (also known as *Ralstonia eutropha* H16) [8]—a metabolically versatile β-proteobacterium with a well-characterized aromatic degradation network—stands out for its capacity to utilize numerous naturally occurring aromatic substrates, including HBAs [9]. The remarkable metabolic flexibility and genetic tractability of *C. necator* H16 have established it as a valuable model for investigating aromatic sensing and as a robust platform for metabolic engineering [10,11,12]. Moreover, synthetic biology increasingly leverages *C. necator* as a chassis for converting renewable feedstocks into valuable chemicals [11,13], resulting in growing interest in exploiting this strain towards sustainable bioproduction.

A deeper understanding of ligand-responsive regulation in *C. necator* and in other hosts can be effectively achieved through biosensor-based approaches [14,15], further advancing metabolic engineering for improved chemical production or application in bioremediation. Many bacteria rely on ligand-responsive inducible gene expression systems that sense intra- or extracellular metabolites and modulate gene expression accordingly. Regulatory sensitivity of system-associated transcription factors (TFs) dictates pathway activation kinetics and overall metabolic flux [16]. By coupling TF activity to fluorescent or otherwise quantifiable reporters, biosensors allow direct, high-resolution monitoring of pathway activation across defined ligand gradients [17]. As a result, TF-based biosensors have become indispensable tools in synthetic biology for pathway optimization, high-throughput screening, and metabolite-responsive circuit engineering [18,19,20]. A detailed characterization of ligand sensitivity—including parameters such as gene activation thresholds (AC_min_) and half-maximal effective concentrations (EC_50_)—can define functional sensing ranges for HBAs, expand rational design of tailored sensing systems, enhance metabolic flux control, and guide engineering strategies to expand the organism’s catabolic capabilities. Although the fundamental principles of TF-based biosensors has been established almost a decade ago [17], many natural systems responding to HBAs lack sensitivity [21] and the design of new biosensors remains challenging [22].

In this study, we determined *C. necator’s* capability to degrade HBAs and dihydroxybenzoic acids (dHBAs) and evaluated the sensitivity of 2-HBA (*Cn*NahR/P*_H16_RS08125_*), 4-HBA (*Cn*PobR/P*_pobA_*), and 3,4-dHBA (*Cn*PcaQ/P*_H16_RS30145_*)-inducible systems using a TF-based biosensor approach. We quantified the AC_min_ and EC_50_ values for these systems and compared their performance to the *Ab*PobR/P*_pobA_* system responding to 3,4-dHBA and other previously described systems sensing HBAs or structurally related compounds from different bacterial hosts.

## 2. Materials and Methods

### 2.1. Chemicals

All chemicals used in this study were as follows: sodium salicylate (cat. no. 71945, Sigma-Aldrich, St. Louis, MO, USA) for 2-HBA analysis, 3-HBA (cat. no. H20008, Sigma-Aldrich, St. Louis, MO, USA), sodium 4-hydroxybenzoate (cat. no. 047887, Fluorochem Limited, Hadfield, UK) for 4-HBA assays, 2,5-dHBA (cat. no. 149357, St. Louis, MO, USA), and 3,4-dHBA (cat. no. B24016, Alfa Aesar, Ward Hill, MA, USA). Stock solutions of the inducers were formulated at 100 mM in DMSO (Eurochemicals, Vilnius, Lithuania) and subsequently subjected to serial dilution in DMSO to achieve the required working concentrations.

### 2.2. Bacterial Strains and Media

The following bacterial strains were used in the study: *E. coli* Top10: F-*mcrA*Δ(*mrr*-*hsdRMS*-*mcrBC*)Φ80*lacZ*ΔM15Δ*lacX*74 *recA1 araD*139 Δ(*araleu*)7697 *galU galK rpsL* (StrR) *endA1 nupG* (Thermo Fisher Scientific, Waltham, MA, USA); and *C. necator* H16: wild-type strain (DSM 428 (DSMZ, Braunschweig, Germany)). For plasmid construction and plasmid propagation, *Escherichia coli* Top10 was used as described in [23]. Bacterial cells were grown in Luria–Bertani (LB) medium (Fisher Scientific, Pittsburgh, PA, USA). When required, chloramphenicol was added to a final concentration of 25 µg/mL for *E. coli* Top10, and 10 µg/mL of gentamicin for *C. necator* H16. Solid LB medium was prepared by adding agar to a final concentration of 16 g/L.

RFP fluorescence reporter assays were performed using the *E. coli* Top10 as a host strain. Fluorescence, absorbance, and HBA consumption assays were performed in LB medium.

### 2.3. Identification of Genes Associated with HBA Catabolism

A search of putative genes responsible for HBA catabolism was conducted in the GenBank database (https://www.ncbi.nlm.nih.gov/, accessed on 15 July 2023) [24], and using information on catabolic pathways provided in the bioinformatics resource Kyoto Encyclopedia of Genes and Genomes (KEGG Pathway Database, www.genome.jp/kegg/pathway.html, accessed on 13 July 2023) [25] and data on enzymes available from the BRaunschweig ENzyme DAtabase (BRENDA, www.brenda-enzymes.org, accessed on 14 October 2023) [26]. Homologous proteins encoded by the target genes were identified in *C. necator* H16, and sequence similarity was determined by using the Basic Local Alignment Search Tool (BLAST, https://blast.ncbi.nlm.nih.gov/Blast.cgi accessed on 14 October 2023).

### 2.4. Plasmid Construction

Plasmids were generated using the NEBuilder HiFi DNA Assembly strategy (New England Biolabs, Ipswich, MA, USA) or restriction enzyme–DNA ligase-based cloning and verified by PCR as well as restriction enzyme analysis. Detailed description of the construction of each plasmid, as well as sequences of oligonucleotide primers can be found in ref. [21]. The names of the new designated biosensors are as follows: *Cn*NahR/P*_H16_RS08125_ *(plasmid pEH042) was designated as the 2-HBA-*Cn*IS biosensor; *Cn*PobR/P*_pobA_* (plasmid pEV021) as 4-HBA-*Cn*IS; *Ab*PobR/P*_pobA_* (plasmid pEV031) as 4-HBA-*Ab*IS; and *Cn*PcaQ/P*_H16_RS30145_* (plasmid pEH161) as 3,4-dHBA-*Cn*IS.

For generating pEH042, oligonucleotide primers EH118_r (5′-ggccggcctggcgcgccataaaacgaaaggctcagtcgaaagactgggcctttcgttttatgacgtcctaatccgaaaacaagccgacg-3′) and EH119_f (5′-gctactcgccatatgtgtctccggctatgtctcttcg-3′) were used to amplify the 2-HBA-inducible system from the genomic DNA of *C. necator* H16, and the resulting DNA fragment was cloned into pBRC1 via AscI and NdeI restriction sites as described in ref. [27]. pEV021 was constructed by using oligonucleotide primers EV056 (5′-gggcctttcgttttatgacgtctaagaaatcgaccaggcaggct-3′) and EV057 (5′-cgtcttcgctactcgccatatgttgtctccttgtggttatcggtc-3′) to amplify 4-HBA-inducible system from genomic DNA of *C. necator* H16, and cloning the resulting DNA fragment into the same vector as above by AatII and NdeI sites. For generating pEV031, oligonucleotide primers EV089 (5′-gggcctttcgttttatgacgtcttataccaaattacgcagctcattcg-3′) and EV090 (5′-cgtcttcgctactcgccatatgatcatccttgctattttctatttttaaagtaccg-3′) were used to amplify the 4-HBA-inducible system using genomic DNA of *A. baylyi* ADP1 as a template, and resulting PCR fragment cloned into pBRC1 vector via AatII and NdeI sites. pEH161 was constructed by using oligonucleotide primers EH289_r (5′-tcgttttatgacgtctcactgagcggaagcagg-3′) and EH290_f (5′-gctactcgccatatggcagtctcctcgtcgttg-3′) to amplify the 3,4-dHBA-inducible system of *C. necator* H16, and cloning the resulting DNA fragment into pBRC1 by AatII and NdeI sites.

### 2.5. Cloning and Transformation

Plasmid DNA was purified using the GeneJET Plasmid Miniprep Kit (Thermo Fisher Scientific, Waltham, MA, USA). Microbial genomic DNA was isolated with the GenElute Bacterial Genomic DNA Extraction Kit (Sigma-Aldrich, St. Louis, MO, USA). For cloning procedures, DNA fragments were amplified in 20 µL PCR employing Phusion High-Fidelity DNA Polymerase (Thermo Fisher Scientific Baltics, Vilnius, Lithuania). Linearized, gel-purified DNA was obtained using the Zymoclean Gel DNA Recovery Kit (ZYMO Research Europe GmbH, Freiburg im Breisgau, Germany). The NEBuilder HiFi DNA Assembly Master Mix (New England Biolabs, Ipswich, MA, USA) was used for DNA assembly. Restriction enzymes were sourced from Thermo Fisher Scientific Baltics (Vilnius, Lithuania), and RapidTaq DNA Polymerase was obtained from Vazyme (Nanjing, China). All PCR, restriction digestion, and DNA assembly reactions were performed according to the respective manufacturers’ instructions.

For cell transformation, *Escherichia coli* Top10 competent cells were combined with plasmid DNA and subjected to a brief cold incubation, a heat-shock step, and a subsequent return to cold conditions, as described previously [23]. Transformed cultures were then allowed to recover in LB medium at 37 °C before being spread onto LB agar plates containing the appropriate antibiotic and incubated overnight at 37 °C.

### 2.6. HBA and dHBA Consumption Analysis Using HPLC-Based Quantification

The consumption of various HBAs and dHBAs by *C. necator* H16, as well as their stability in LB medium-only, was analyzed by inoculating bacterial cells in 2 mL of LB medium, and incubating overnight at 30 °C and 200 rpm. The cultures were afterwards inoculated into 7 mL of fresh medium at a ratio of 1:50 in 50 mL tubes and incubated under the same conditions until OD_600_ reached 0.1–0.2. Then, HBAs to be tested were added parallelly to the cultures and the fresh LB medium up to a final concentration of 5 mM. A volume of 150 µL of samples was taken immediately, and then repeatedly at 1, 3, 6, and 24 h after adding the respective HBA. The OD_600_ was observed at each time point for *C. necator* cultures. For LB medium-only, samples were taken at 0 h and 24 h of the same volume. Finally, the samples were centrifuged for 5 min and 14,000 rpm. The supernatant was collected and diluted with distilled water at a ratio of 1:10 and subjected to HPLC analysis.

The diluted samples were filtered with 0.22 µm pore size membrane syringe filters. The samples were analyzed using the Ultimate 3000 HPLC system (Thermo Fisher Scientific, Waltham, MA, USA) using a Luna C18 column (5 μm, 100 Å, 150 × 4.60 mm) (Phenomenex, Torrance, CA, USA), equipped with the Security Guard Cartridge (part number KJ0-4282, Phenomenex, Torrance, CA, USA) and maintained at 25 °C. The mobile phase consisted of solvent A (0.1% formic acid in water, *v/v*) and solvent B (HPLC-grade acetonitrile). The gradient elution program was as follows: 10% to 50% B from 0 to 15 min; 50% to 70% B from 15 to 17.5 min; 70% to 10% B from 17.5 to 20 min, followed by an isocratic hold at 10% B for 2 min. The flow rate was 1 mL/min with a 10 μL injection volume. Each HBA was detected at 320 nm and identified by comparing retention times with those of standards. Samples were run for 30 min. Chromatograms were processed using Chromeleon 7 software (Thermo Fisher Scientific, Waltham, MA, USA). The concentrations of 2-HBA, 3HBA, 4-HBA, 2,5-dHBA and 3,4-dHBA were estimated from standard curves generated by analyzing known quantities of these compounds.

### 2.7. Fluorescence and Absorbance Measurements

Reporter signal intensity and culture optical density were monitored over time in a plate reader Infinite^®^ M Nano+ microplate reader (Tecan, Grödig, Austria). Bacterial strains were first grown overnight in 2 mL LB medium with the respective antibiotic, at 200 rpm and 30 °C. Overnight cultures were diluted to an OD_600_ of 0.05 in fresh medium with appropriate antibiotic, and grown to an OD_600_ of 0.15–0.2. Cultures measuring 142.5 µL in volume were then transferred to 96-well plates (flat and clear bottom, black; Corning Incorporated, Corning, NY, USA) and 7.5 μL of the inducer of the respective concentration was added up to a total volume of 150 μL. Fluorescence measurements were collected every 10 min over a period of 15 h, with an excitation wavelength of 585 nm and emission wavelength of 620 nm. In parallel, absorbance was measured at 600 nm. The fluorescence gain factor was set to 120%.

Fluorescence data were quantitatively normalized by calculating the absolute normalized fluorescence (ANF), yielding a standardized metric for comparing reporter output across experimental conditions, as previously described [27]:

(1) $$ANF=\;\frac{{RFP}_{raw}\;-\;{RFP}_{medium}}{{OD}_{raw}\;-\;{OD}_{medium}}$$

where *RFP_raw_* and *OD_raw_* represent the absolute fluorescence and absorbance values of the culture, while *RFP_medium_* and *OD_medium_* correspond to the fluorescence and absorbance values of the medium alone.

Relative normalized fluorescence *RNF*(%) values were obtained by using ANF values at a specific inducer concentration:

(2) $$RNF(\%)=100\;\times \;\frac{\;ANF\;-\;b_{min}}{b_{max}}$$

where ANF is absolute normalized fluorescence; *b_max_* and *b_min_* represent the maximum and minimum reporter outputs in ANF units, respectively.

### 2.8. Evaluation of AC_min_ and EC_50_

The dose–response was determined as previously described [21] using values of fluorescence and absorbance 6 h after extracellular addition of the respective compound, in the concentration range from 0.61 µM to 5 mM. The assays were performed as described above in 96-well plates, using the same conditions.

The EC_50_ was calculated using Formula (4), derived from the Hill function (3) [28], with GraphPad Prism 9 software:

(3) $$ANF\;(I)=b_{max}\times \frac{I^{h}}{{EC}_{50}+I^{h}}+b_{min}$$

(4) $${EC}_{50}=I\times {\left(\frac{b_{max}}{ANF\;(I)-b_{min}}-1\right)}^{\frac{1}{h}}$$

where *ANF (I)* denotes the *ANF* value at a given ligand concentration *I*; *b_max_* and *b_min_* represent the maximum and minimum reporter outputs in *ANF* units, respectively; *h* is the Hill coefficient; and EC_50_ is the ligand concentration that yields half-maximal reporter output.

AC_min_ was derived from the dose–response relationship, in which reporter output was quantified as a function of inducer concentration. AC_min_ represents the minimum concentration of ligand required to trigger a response and it is defined as the lowest ligand concentration that produced a reporter’s signal statistically greater than the next lower concentration and typically greater than all lower concentrations.

AC_min_ and EC_50_ were derived using a single time point measured 6 h after extracellular addition of the respective compound. The 6 h time point was used for parameter estimation ensuring that all cells are in the exponential growth phase and are able to exhibit active metabolism. It should be noted that minor variations in these parameters can be observed when alternative time points are used for analysis.

### 2.9. Statistical Analysis

All data in this study are reported as mean ± SD (*n* = 3). Statistical analyses were performed in GraphPad Prism 9.0 using an unpaired two-tailed *t*-test to compare group means. *p*-values of 0.05, 0.01, or 0.001 were considered statistically significant.

## 3. Results and Discussion

### 3.1. Metabolism and Consumption of HBAs and dHBAs in C. necator

**Figure 1:**
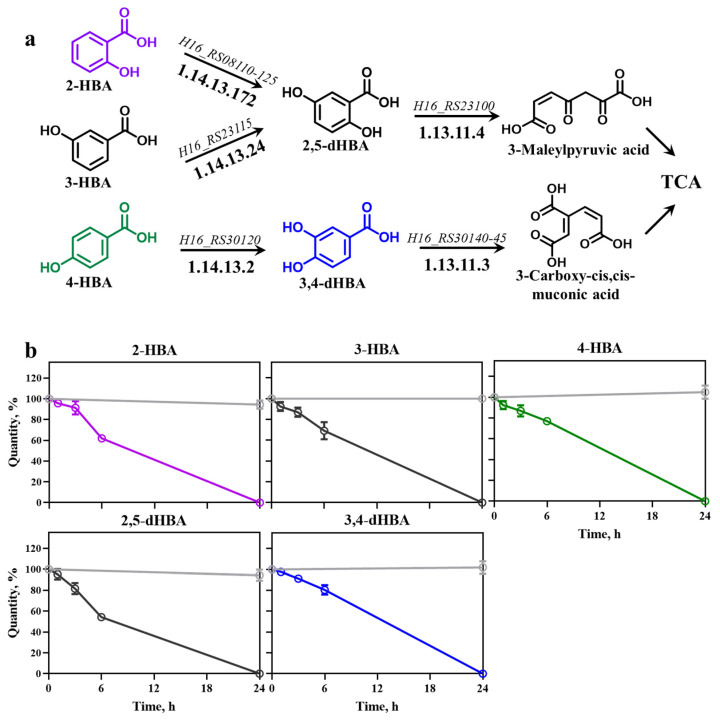
HBA metabolism and consumption in *C. necator* H16. (**a**) Metabolic pathways occurring in *C. necator*; (**b**) consumption of HBAs and dHBAs, including 2-HBA, 3-HBA, and 4-HBA, 2,5-dHBA, and 3,4-dHBA in *C. necator* (curves highlighted in color corresponding to the coloring chemical structures in (**a**)) compared with their stability in LB medium (light gray), determined with HPLC. Each HBA and dHBA was added at 0 h at a final concentration of 5 mM. Error bars represent standard deviation of three biological replicates.

HBAs and dHBAs are known to be effectively metabolized in certain bacteria [21]. Given that the *Burkholderiales* order is rich in genes encoding HBA-catabolizing enzymes [1,29], we identified genes and related enzymes responsible for catabolic entry reactions of HBAs and dHBAs in *C. necator* H16 using information from databases such as KEGG [25] and BRENDA [26]. In this bacterium, 2-HBA, 3-HBA, and 4-HBA is converted to 2,5-dHBA or 3,4-dHBA by salicylate hydroxylase (*H16_RS08110-125*), 3-hydroxybenzoate 6-monooxygenase (*H16_RS23115*), and 4-hydroxybenzoate 3-monooxygenase (*H16_RS30120*), respectively (Figure 1a). Subsequently, 2,5-dHBA’s and 3,4-dHBA’s aromatic rings are cleaved by gentisate 1,2-dioxygenase (*H16_RS23100*) and protocatechuate 3,4-dioxygenase (*H16_RS30140-45*), and resulting metabolic intermediates 3-methylpyruvic acid and 3-carboxy-cis, cis-muconic acid, respectively, are directed towards the TCA cycle.

To confirm the ability of *C. necator* H16 to catabolize HBAs, we assessed the consumption of mono- and di-HBAs. HBAs were added to exponentially growing cells and samples were taken at 0, 1, 3, 6, and 24 h to monitor change in HBA concentration as described in Section 2.

All tested HBAs remained stable in cell-free control samples over the entire 24 h period (Figure 1b). In contrast, *C. necator* H16 efficiently catabolized 2-HBA, 3-HBA, 4-HBA, 2,5-dHBA, and 3,4-dHBA. Specifically, 2-HBA was reduced by ~60% after 6 h and completely depleted by 24 h. Similarly, 3-HBA remained stable in LB medium but was reduced to ~70% at 6 h and fully consumed after 24 h by *C. necator*. Likewise, 2,5-dHBA and 3,4-dHBA were stable in LB medium over 24 h. In *C. necator*, 2,5-dHBA decreased by ~50% at 6 h and was fully catabolized by 24 h, whereas 3,4-dHBA showed ~80% depletion at 6 h and complete consumption by 24 h (Figure 1b). These findings demonstrate that *C. necator* possesses highly efficient catabolism of HBA, likely at the entry point driven by genes and corresponding enzymes identified in Figure 1a.

### 3.2. Design of TF-Based HBA Biosensors

**Figure 2:**
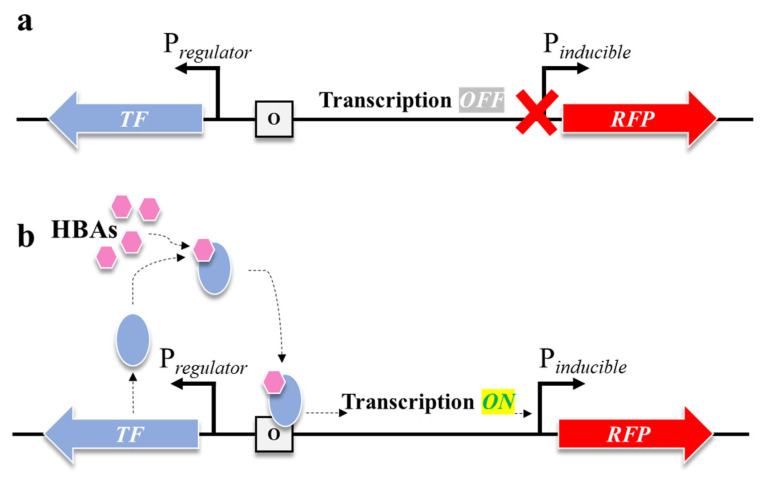
Schematic illustration of a TF controlling expression of inducible genes. (**a**) In the absence of a ligand molecule (HBA), the transcription state is *OFF* and the synthesis of downstream gene-encoding proteins is not occurring. (**b**) In the presence of HBA, the TF connects to it, making a complex and initiating the action of RNA polymerase (transcription *ON*), where the downstream pathway gene-encoding protein is synthesized and RFP signal increases.

TF-based biosensor is typically composed of a reporter (fluorescence reporter in this study) and an inducible system that controls the gene expression in response to defined external or endogenous signal [15]. Although inducible systems vary widely in their molecular architecture, they generally share three fundamental components: a ligand molecule, a TF, and an inducible promoter (Figure 2) [17].

In this study, we assessed the sensitivity of HBA- and dHBA-inducible systems from *C. necator*. Previously described 2-HBA (*Cn*NahR/P*_H16_RS08125_*), 4-HBA (*Cn*PobR/P*_pobA_*), and 3,4-dHBA (*Cn*PcaQ/P*_H16_RS30145_*) inducible systems [21] were used for the construction of TF-based biosensors. The ligand molecules 2-HBA, 4-HBA, and 3,4-dHBA interact specifically with their cognate TFs NahR, PobR, and PcaQ, respectively. This interaction is highly selective and typically mediated through a well-defined ligand-binding domain, resulting in a new conformational state of the TF [30]. The TF–HBA complex binds to a specific operator upstream of the regulated promoter. This interaction increases the probability that RNA polymerase (RNAP) will initiate a transcription of the downstream gene, which is replaced with the red fluorescent protein (RFP) in the biosensor. Once the promoter is activated, the target gene is transcribed, producing mRNA and ultimately the encoded protein (Figure 2b). The magnitude and kinetics of expression typically correlate with the concentration of effector molecule, enabling dose-dependent control [28]. The TFs of the HBA- and dHBA-inducible systems from *C. necator*, namely NahR, PobR, and PcaQ, belong to the LysR [31] and AraC/XylS [32] families, respectively, and act as activators or dual-function TFs [21]. Despite originating from distinct TF families, NahR, PobR, and PcaQ converge on a similar regulatory mechanism; they operate through a comparable mechanism of ligand-dependent promoter activation in *C. necator*.

### 3.3. Determination of AC_min_ and EC_50_ of HBA Biosensors

**Figure 3:**
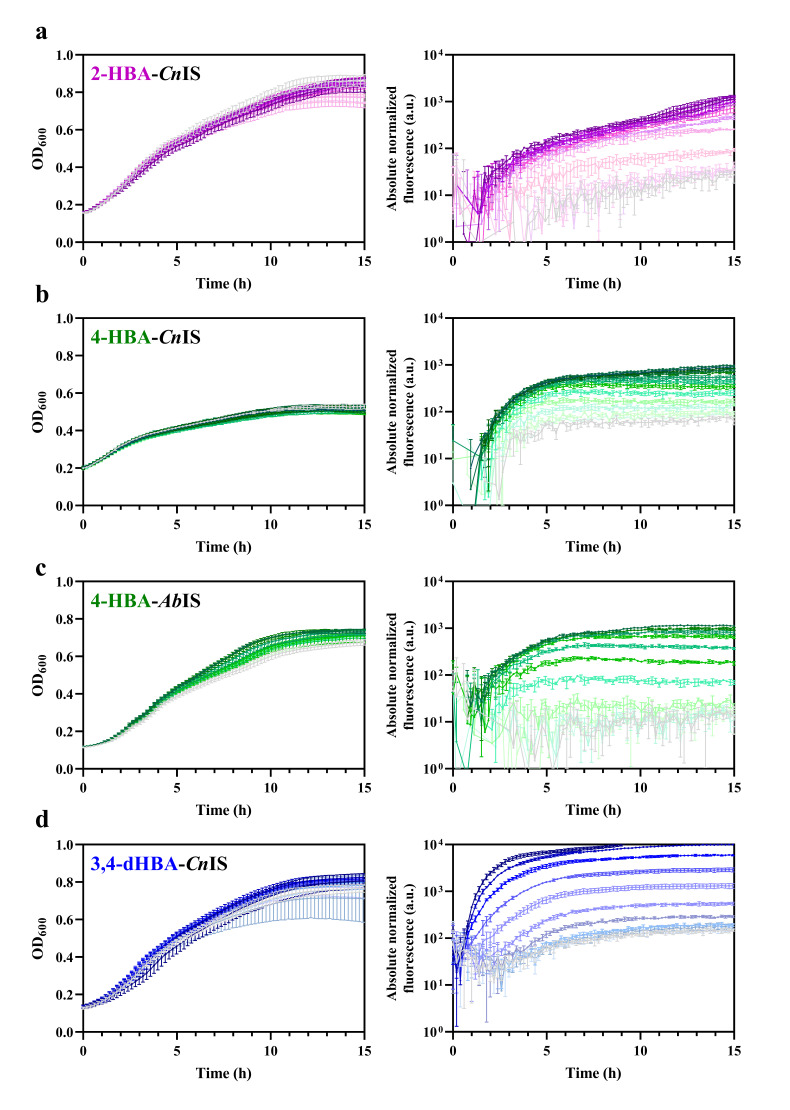
Cell growth dynamics and induction kinetics of the HBA and dHBA biosensors. Graphs represent the optical density over time and absolute normalized fluorescence of (**a**) 2-HBA-*Cn*IS, (**b**) 4-HBA-*Cn*IS, (**c**) 4-HBA-*Ab*IS, and (**d**) 3,4-dHBA-*Cn*IS biosensors. Cells were grown in LB medium for 15 h. Absorbance and fluorescence were measured in the absence of inducer (light gray) and in the presence of different concentrations of the corresponding HBA, ranging from the lowest non-inducing concentration defined in Figure 4 up to 0.625 mM (2-HBA-*Cn*IS), 2.5 mM (4-HBA-*Cn*IS), or 5 mM (4-HBA-*Ab*IS and 3,4-dHBA-*Cn*IS), the darker shades indicate higher concentrations. Error bars represent standard deviation of three biological replicates. Absolute normalized fluorescence is displayed in the logarithmic scale. Dose–response curves, obtained by plotting RNF (%) against inducer concentration measured 6 h after HBA addition as described previously [21], were used to determine EC_50_ and AC_min_ (Table 1 and Figure 4) as described in Section 2. The EC_50_ value represents the ligand concentration that produces half-maximal reporter’s output. This parameter was extracted from the dose–response curves by fitting the data using the Hill function. The AC_min_ corresponded to the lowest ligand concentration that yielded a reporter signal distinguishably higher than responses observed at the next lower and, typically, all lower concentrations. Statistical analysis confirmed the significance of this increase.

To determine the sensitivity of the HBA biosensors derived using inducible gene expression systems from *C. necator* and to compare them to the *A. baylyi* system, constructs carrying *Cn*NahR/P*_H16_RS08125_* (plasmid construct pEH042), *Cn*PobR/P*_pobA_* (pEV021), *Ab*PobR/P*_pobA_* (pEA031), and *Cn*PcaQ/P*_H16_RS30145_* (pEH161) described in [21] were used. Introduction of these systems into *E. coli* Top10 resulted in the corresponding 2-HBA-*Cn*IS, 4-HBA-*Cn*IS, 4-HBA-*Ab*IS, and 3,4-dHBA-*Cn*IS biosensors. *E. coli*-based biosensors were grown in LB medium, and reporter gene expression was monitored after extracellular supplementation with the corresponding HBA or dHBA, as indicated in Table 1, across a concentration range of 0–5 mM (Figure 3).

**Figure 4:**
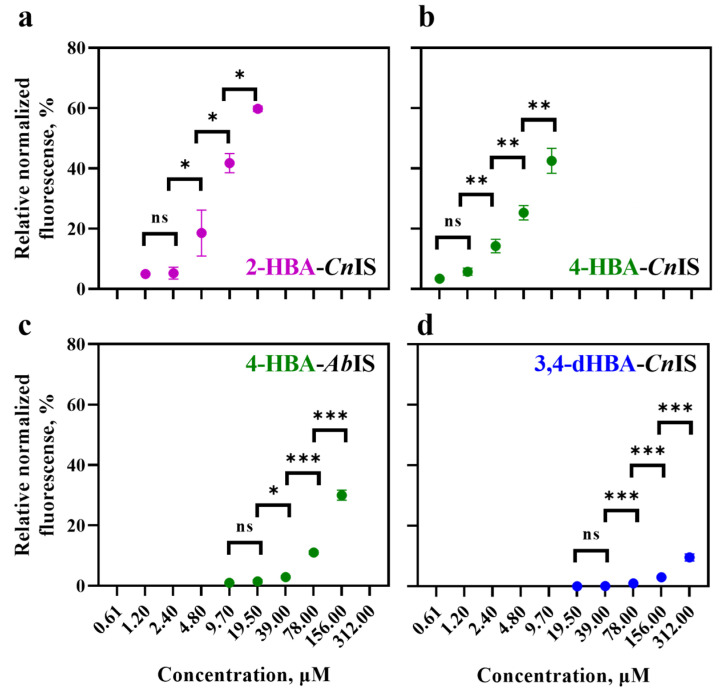
Relative normalized fluorescence values (%) at a single time point for *E. coli* Top10 strains carrying HBA and dHBA biosensors: (**a**) 2-HBA–*Cn*IS (supplemented with 2-HBA), (**b**) 4-HBA–*Cn*IS (supplemented with 4-HBA), (**c**) 4-HBA–*Ab*IS (supplemented with 4-HBA), and (**d**) 3,4-dHBA–*Cn*IS (supplemented with 3,4-dHBA). Fluorescence output was measured 6 h after extracellular addition of the respective compound. Error bars represent the standard deviation of three biological replicates. Statistically significant difference is indicated by asterisks: * *p* < 0.05, ** *p* < 0.01, *** *p* < 0.001 (unpaired two-tailed *t*-test), and "ns" shows that the difference is not significant.

**Figure 5:**
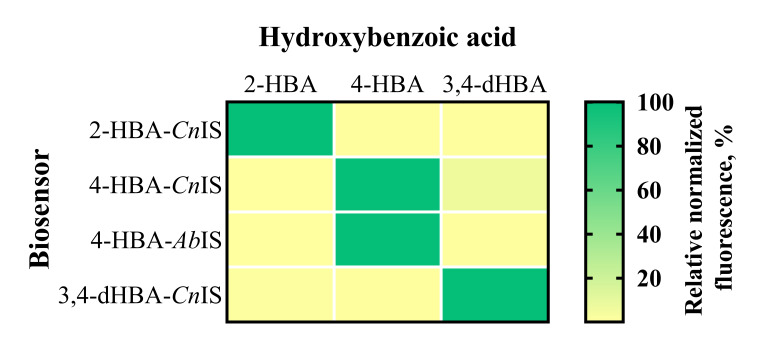
Specificity and cross-reactivity of HBA and dHBA biosensors. The heat map shows responses of biosensors in the presence of 2-HBA, 4-HBA, and 3,4-dHBA, acting as non-specific ligands or primary effectors. The response is expressed in % relative to the biosensor’s activation by the primary effector. Fluorescence output was measured 6 h after extracellular addition of HBA at a final concentration of 5 mM. Data are mean, *n* = 3.

The *E. coli*-based 2-HBA-*Cn*IS and 4-HBA-*Cn*IS biosensors, containing the *Cn*NahR/P*_H16_RS08125_* and *Cn*PobR/P*_pobA_* inducible systems, respectively, exhibited low AC_min_ values (4.8 μM and 2.4 μM) and EC_50_ values (19.91 μM and 13.06 μM) in response to 2-HBA and 4-HBA, respectively (Table 1, Figure 3 and Figure 4). For comparison, the 4-HBA-*Ab*IS biosensor, carrying a 4-HBA-inducible system homologous to the *C. necator Cn*PobR/P*_pobA_* system, showed substantially higher AC_min_ and EC_50_ values of 39.0 μM and 280.47 μM, respectively (Table 1, Figure 3 and Figure 4). Additionally, we evaluated the 3,4-dHBA–*Cn*IS biosensor, which carries the *Cn*PcaQ/P*_H16_RS30145_ *inducible system originating from *C. necator*, in response to 3,4-dHBA and obtained an AC_min_ value of 78 μM (Table 1, Figure 3 and Figure 4). The low AC_min_ and EC_50_ values of the *Cn*NahR/P*_H16_RS08125_* and *Cn*PobR/P*_pobA_* systems indicate efficient 2-HBA and 4-HBA recognition and strong inducible responses at micromolar concentrations. In contrast, the homologous 4-HBA-*Ab*IS biosensor required substantially higher 4-HBA concentrations to achieve activation, highlighting the superior sensitivity and performance of the *C. necator*-derived regulatory elements in *E. coli*. It should be noted that insignificant level of cross-reactivity was observed between the non-specific ligands and the biosensors 2-HBA-*Cn*IS, 4-HBA-*Ab*IS, and 3,4-dHBA-*Cn*IS compared to the responses observed when these sensors were subjected to primary effectors (Figure 5). Only 4-HBA-*Cn*IS exhibited a minor non-specific activation by 3,4-dHBA. As *E. coli* does not possess metabolic pathways for transformation of either 4-HBA or 3,4-dHBA, it can be concluded that observed cross-reactivity can be attributed to a structural resemblance of these two compounds.

### 3.4. Comparison of AC_min_ and EC_50_ Values of HBA Biosensors Across Different Bacterial Hosts

A cross-system comparison shows that the *C. necator* H16 regulators characterized in this study—*Cn*PobR and *Cn*NahR—perform similarly to other previously reported systems from *Pseudomonas* and *Comamonas* species, all belonging to *Burkholderiales* order [21,33,34] (Table 2). Both *Cn*PobR/P*_pobA_* and *Cn*NahR/P*_H16_RS08125_* systems from *C. necator* exhibit low-micromolar activation thresholds (2.4 and 4.8 µM), placing them among the most sensitive native systems responsive to 4-HBA and 2-HBA described to date. Similarly, the previously reported benzoic acid (BA)-inducible system from *C. necator* also showed low AC_min_ and EC_50_ values of approximately 1 μM and 12.6 µM, respectively [27]. In addition, the *Cn*PaaX system was shown to display an even lower AC_min_ value of 0.00239 µM [35,36].

Comparison of the 4-HBA-responsive system from *C. necator* with the analogous *A. baylyi* system in this study revealed that the *C. necator* variant exhibits 16-fold lower AC_min_ and 21.5-fold lower EC_50_ values. In combination, these results showed that *C. necator* possess BA- and mono HBAs-inducible systems exhibiting high ligand sensitivity. Our findings suggest that this chemolithoautotrophic bacterium evolved sensing systems based on the gene regulatory mechanisms to detect and scavenge for simple aromatic compounds. Systems’ high sensitivity and ability to respond at low micromolar concentrations of the ligand further supports their suitability for applications aimed at monitoring or optimizing aromatic compound uptake and conversion.

In contrast, *Cn*PcaQ, while functional towards 3,4-dHBA, exhibited a significantly higher AC_min_ (78 µM) compared to *Cn*NahR- and *Cn*PobR-based systems, indicating that dHBA-inducible regulators are considerably less sensitive than those responsive to HBAs. Despite that the fundamental principles, mainly defined by the strength of the interaction between ligand and TF, or/and TF and operator, are described at a theoretical level [17], further research is required to validate the molecular mechanism by which the high ligand sensitivity is determined in *Cn*NahR and *Cn*PobR systems and corresponding biosensors 2-HBA-*Cn*IS and 4-HBA-*Cn*IS.

## 4. Conclusions

In this study, by applying a TF-based biosensor approach, we quantified how efficiently *C. necator* can activate its hydroxybenzoate catabolic regulons in response to extracellular HBAs. The three systems examined—responding to 2-HBA, 4-HBA, and 3,4-dHBA—displayed distinct sensing profiles, with the 2-HBA- and 4-HBA-responsive regulators exhibiting particularly low AC_min_ (4.8 μM and 2.4 μM, respectively) and correspondingly low EC_50_ (19.91 μM and 13.06 μM, respectively) values. These results indicate that *C. necator* deploys highly sensitive inducible gene expression systems for monohydroxybenzoate degradation, consistent with a metabolism geared toward efficient scavenging of low-abundance aromatics in the environment.

Beyond clarifying how *C. necator* prioritizes and commits to aromatic catabolism, the findings can help leverage native inducible systems for selective detection, pathway analysis, and broader biotechnological applications. Moreover, the TF-based biosensor approach applied for mapping functional sensing ranges in combination with research and informatics of catabolism can be used to guide metabolic engineering efforts aimed at expanding substrate utilization spectra, tuning pathway activation dynamics, and anticipating substrate-competition effects in complex mixtures.

## Figures and Tables

**Table 1:** Parameters of HBA and dHBA biosensors. AC_min_ and EC_50_ were derived using a single time point measured 6 h after extracellular addition of the respective compound. Data are mean ± SD, *n* = 3.

Biosensor	Ligand	Inducible System	Microbial Chassis	AC_min_, μM	EC_50_, μM
2-HBA-*Cn*IS	2-HBA	*Cn*NahR/P*_H16_RS08125_*	*E. coli* Top10	4.8	19.91 ± 6.94
4-HBA-*Cn*IS	4-HBA	*Cn*PobR/P*_pobA_*	*E. coli* Top10	2.4	13.06 ± 2.49
4-HBA-*Ab*IS	4-HBA	*Ab*PobR/P*_pobA_*	*E. coli* Top10	39	280.47 ± 12.99
3,4-dHBA-*Cn*IS	3,4-dHBA	*Cn*PcaQ/P*_H16_RS30145_*	*E. coli* Top10	78	1257.67 ± 65.24

**Table 2:** Comparison of HBAs and structurally related compound biosensors based on AC_min_ and EC_50_ parameters.

HBAs and Structurally Related Compounds	Inducible System	Source	Host	AC_min_, μM	EC_50_, μM	Reference
2-HBA	*Pp*NahR/Psal/Pr	*P. putida*	*E. coli*	80	~300–500	[33]
3-HBA	*Ct*MobR/P*_mobA_*	*C. testosteroni* ATCC 11996	*P. putida* KT2440	~300	2894	[21]
3,4-dHBA	*Pp*PcaU/P*_PcaU1.2_*	*P. putida* KT2440	*P. putida*	~10	~70–90	[34]
BA	*Cn*BenM/P*_H16_RS09790_*	*C. necator* H16	*C. necator* H16	~1	12.6	[27]
Phenylacetic acid	*Cn*PaaX/P*_paaA2_*	*C. necator* H16	*C. necator* H16	0.00239	0.0390	[36]
2,5-dHBA	*Cn*GtdR/P*_gtdA_*	*C. necator* H16	*E. coli*	0.1	0.00952	[35]
2-HBA	*Cn*NahR/P*_H16_RS08125_*	*C. necator* H16	*E. coli*	4.8	19.91	this work
4-HBA	*Cn*PobR/P*_pobA_*	*C. necator* H16	*E. coli*	2.4	13.06	this work
4-HBA	*Ab*PobR*/*P*_pobA_*	*A. baylyi*	*E. coli*	39	280.47 ± 12.99	this work
3,4-dHBA	*Cn*PcaQ/P*_H16_RS30145_*	*C. necator* H16	*E. coli*	78	1257.67	this work

## Data Availability

The raw data supporting the conclusions of this article will be made available by the authors on request.

## Abbreviations

The following abbreviations are used in this manuscript:

2-HBA	2-hydroxybenzoic acid
3-HBA	3-hydroxybenzoic acid
4-HBA	4-hydroxybenzoic acid
2,5-dHBA	2,5-dihydroxybenzoic acid
3,4-dHBA	protocatechuic acid
TCA	tricarboxylic acid
TF	transcription factor
AC_min_	gene activation threshold
EC_50_	half-maximal effective concentration
HBAs	hydroxybenzoic acids
dHBAs	dihydroxybenzoic acids
ANF	absolute normalized fluorescence
RNF	relative normalized fluorescence
